# Oxidation Protection in Metal-Binding Peptide Motif and Its Application to Antibody for Site-Selective Conjugation

**DOI:** 10.1371/journal.pone.0159451

**Published:** 2016-07-15

**Authors:** Hye-Shin Chung, Sunbae Lee, Soon Jae Park

**Affiliations:** 1 Department of Biological Sciences and Biotechnology, College of Life Science and Nano Technology, Hannam University, 1646, Yuseong-daero, Yuseong-gu, Daejeon, Korea; 2 Alteogen Inc., Yuseong-daero 62, Jeon-min Dong, Yuseong-gu, Daejeon, Korea; University of Akron, UNITED STATES

## Abstract

Here, we demonstrate that a metal ion binding motif could serve as an efficient and robust tool for site-specific conjugation strategy. Cysteine-containing metal binding motifs were constructed as single repeat or tandem repeat peptides and their metal binding characteristics were investigated. The tandem repeats of the Cysteine-Glycine-Histidine (CGH) metal ion binding motif exhibited concerted binding to Co(II) ions, suggesting that conformational transition of peptide was triggered by the sequential metal ion binding. Evaluation of the free thiol content after reduction by reducing reagent showed that metal-ion binding elicited strong retardation of cysteine oxidation in the order of Zn(II)>Ni(II)>Co(II). The CGH metal ion binding motif was then introduced to the C-terminus of antibody heavy chain and the metal ion-dependent characteristics of oxidation kinetics were investigated. As in the case of peptides, CGH-motif-introduced antibody exhibited strong dependence on metal ion binding to protect against oxidation. Zn(II)-saturated antibody with tandem repeat of CGH motif retains the cysteine reactivity as long as 22 hour even with saturating O_2_ condition. Metal-ion dependent fluorophore labeling clearly indicated that metal binding motifs could be employed as an efficient tool for site-specific conjugation. Whereas Trastuzumab without a metal ion binding site exhibited site-nonspecific dye conjugation, Zn(II) ion binding to antibody with a tandem repeat of CGH motif showed that fluorophores were site-specifically conjugated to the heavy chain of antibody. We believe that this strong metal ion dependence on oxidation protection and the resulting site-selective conjugation could be exploited further to develop a highly site-specific conjugation strategy for proteins that contain multiple intrinsic cysteine residues, including monoclonal antibodies.

## Introduction

Site-selective conjugation at proteins has been widely employed to detect cellular distribution, molecular interactions, cellular trafficking, and changes in mobility or conformation of proteins [1–5]. Many researchers have developed sophisticated conjugation technologies to label payloads at the desired target sites in proteins [2,6,7]. Amine and cysteine have been the major targets in conjugation chemistry [8]. Lysine or arginine are relatively abundant in proteins, so amine conjugation eventually leads to site-nonspecific conjugation. Cysteine residues are usually scarce in protein, and cysteine conjugation methods are well established, generally having a high conjugation yield. However, the presence of intrinsic cysteine residues in some proteins complicates site-selective conjugation if those residues are not the ideal conjugation target. Moreover, intrinsic cysteine residues are usually present in disulfide bonds, sustaining the protein’s conformational stability [9,10]. Therefore, labeling a fluorophore or other hydrophobic moiety at these positions might compromise overall protein stability, and conjugation with a small molecule can significantly decrease the enzymatic activity [7,11]. Even if not associated with a disulfide bridge, labeling at cysteine residues that might be involved in electrostatic or hydrophobic interactions essential for secondary or tertiary structural conformation could be problematic [12]. To avoid these issues, intrinsic cysteines are often mutated into other amino acids [13], and other cysteine residues are introduced into a specific target position that does not perturb the original protein conformation. In such cases, silencing the intrinsic cysteine residues might be still problematic. Accordingly, the best alternative is introducing a cysteine residue in a specific target position while still retaining the original cysteine residue, in case there is means to differentiate the reactivity of the introduced cysteine from the intrinsic cysteine. Here, we present data indicating that the metal ion chelating peptide motif could be utilized for that purpose.

Metal ions play a central role in diverse cellular processes and living organisms have developed their own sensing, trafficking, or sequestering mechanisms to maintain the homeostasis of intracellular metal ion concentrations [14,15]. The zinc finger motif is one of the best known mechanisms utilized by various proteins to sustain structural integrity. Two cysteines and two histidines comprise the metal binding motif in the C2H2 group of the zinc finger family, and zinc-coordination induced finger-like small protein motifs are commonly found in mammalian transcription factors [16,17]. Transcriptional regulatory proteins such as the ArsR/SmtB family regulate transcription of the czr operon in response to Zn(II) and Co(II) in vivo [18]. Additionally, metal ion binding in this family triggers a quaternary conformation change that regulates the binding affinity of these proteins to the target DNA region [19]. Cys motifs such as Cys-X-X-Cys or Cys-X-Cys are frequently found in transcriptional regulatory proteins, metal chaperone proteins, and metal ion transporters [20,21]. Cysteines have attracted a great deal of interest in the field of de novo metallopeptide design due to the high affinity of thiols for heavy metal ions such as Hg(II), Cd(II), Bi(II), and Pb(II) [22,23]. Mutationally incorporated tri-cysteine motif at the C-terminal end of α3DIV enabled the mutated proteins to bind toxic heavy metal ions with binding constants as low as 2~3 × 10^7^ M^-1^ for Pb(II) and Cd(II) [24].

De novo designed metal binding motifs usually have tetra- or tri-peptide structures. Square planar or tetrahedral coordination environments can easily be achieved with tri- or tetra-peptide structures [25, 26], providing a flexible and stable chelate ring for metal ions. Burrows et al. prepared a CGH motif and characterized its coordination chemistry [27]. They showed that the CGH motif binds to Ni(II) with square planar coordination, and, interestingly, that coordination behavior exhibited pH-dependency. At pH values over 8.5, Ni(II) is coordinated to four alpha nitrogens, while it is coordinated to one thiol and three alpha nitrogens at lower pH. They also found that cysteine residues in CGH motifs are strongly protected from air oxidation with Zn(II) coordination at neutral pH, while Ni(II)- or Zn(II)-complexed peptide was protected from air oxidation for as much as 40 hours. This cysteine protection capability of metal ion is worth cultivating, and metal-ion dependent oxidation protection could be exploited as a site-selective conjugation strategy for proteins.

Here, we propose that the metal-ion binding motif could be exploited for site selective labeling strategy. Site-selective conjugation utilizing metal-binding motifs have many advantages over other alternatives. For example, cysteine reactivity in metal binding motifs can be easily controlled by the presence of metal ions [28,29]. Metal binding to a short metal binding motif is a reversible reaction, and the bound metal ions can easily be removed simply by the addition of high affinity chelating agent, such as EDTA. Moreover, relatively short tri-, tetra-, or penta-peptide motifs could easily be incorporated into the protein without disturbing its structural stability. If it is necessary to introduce multiple conjugation sites, tandem repeats of metal binding motifs could confer additional flexibility in conjugation sites and conjugation number. Here, we investigated the metal binding characteristics and oxidation protection of CGH motif in the formats of single and tandem repeats of peptides. Additionally, the CGH metal binding motif was introduced into antibody and metal ion dependent oxidation protection and site-selective conjugation were also investigated. Specifically, trastuzumab, a monoclonal antibody that selectively binds to *Her*2 receptors, was used to test the conjugation selectivity. We believe that introduction of the metal binding motif into the antibody could provide an efficient platform for site-specific conjugation sites.

## Materials and Methods

### 2.1 Peptide preparation

N-terminal acetylated M1 peptide (ACGHA) and M2 peptide (ACGHAACGHA) were purchased from Peptron Incorporation (Daejeon, Korea). Alexfluor488-maleimide was obtained from Invitrogen (USA) and used as received.

### 2.2 Construction of antibody variant bearing the metal binding motif

The expression vector was cloned using a pAV4 vector constructed and modified from the parent vector, pSGHV0 (GenBank Accession No. AF285183). To construct a trastuzumab vector, the cDNAs of the heavy chain and light chain of trastuzumab were synthesized and cloned into the XhoI/NotI and ApaI/SmaI restriction sites of the pAV4 vector, respectively. The double repeat of the metal binding motif, ACGHAACGHA, was introduced into the trastuzumab vector as follows. PCR amplification was performed using the trastuzumab vector as a template together with a XhoHH forward primer (5´-GGG GGG CTC GAG ACC ATG GGT TGG AGC TGT -3´) and a M2 reverse primer (5’ CCCCGC GGC CGC CTA GGC ATG GCC ACA AGC AGC ATG GCC ACA GGC GCC GGG AGA CAG AGA 3’). The amplified nucleotide was cleaved with the restriction enzymes XhoI and NotI and ligated with the expression vector pHHL002 having XhoI/NotI cleavage sites, thereby constructing a trastuzumab antibody variant vector (HHL002M2) with an introduced metal binding motif.

### 2.3 Antibody production and purification

The metal-binding motif-introduced trastuzumab antibody variant (HM2) was expressed by inoculating the expression vector HHL002M2 into Chinese hamster ovary cells (CHO-K1). Specifically, CHO-K1 cells were cultured in DMEM (Dulbecco’s Modified Eagle Media) containing 10% FBS (fetal bovine serum) and antibiotic. The CHO-K1 cells were cultured in a 100 mm culture dish at a concentration of 5 × 10^6^ cells/ml, then cultured for 24 hours. Next, 800 μl of FBS- and antibiotics-free DMEM was mixed with 10 μg vector and incubated at room temperature for 1 minute, after which the mixture was mixed with 20 μg polyethylenimine (Polysciences Inc, MW ~25,000) and allowed to stand at room temperature for about 10–15 minutes. Meanwhile, cells were washed with PBS, after which 6 ml of fresh DMEM were added. The HHL002M2 vector was held at room temperature for 10–15 minutes, then added to the culture dish. On the next day, cells were washed with PBS, after which FBS-free IMDM medium (Gibco, Iscove´s Modified Dulbecco´s Medium) was added to confirm the expression of the antibody protein.

The trastuzumab antibody variant HM2, expressed as described above, was purified in the following manner. HM2 was secreted into the cell culture media and harvested on day 4 after IMDM media replacement. The culture medium was then centrifuged to remove cells, after which the supernatant was injected into a HiTrap Protein A HP column (GE Healthcare, USA) that had been equilibrated with equilibration buffer (5 mM histidine, 20 mM arginine, 0.4 M NaCl, pH 6.0). The column was sufficiently washed with equilibration buffer, after which the protein was eluted by altering the pH with citrate buffer (5 mM citrate, 20 mM arginine, pH 3.5). The resulting solution was then dialyzed against phosphate buffer (20 mM sodium phosphate, 0.3 M NaCl, pH 7.4) and concentrated using Vivaspin20 (Sartorius, USA).

### 2.4 DTNB assay for measuring free thiol

A UV-absorption spectrometer was employed to measure the release of TNB through Ellman’s reaction [30]. The free cysteine level in solution is measured by monitoring the change in absorbance at 412 nm by the release of TNB (2-nitro-5-chlorobenzaldehyde) from DTNB (5,5’-dithiobis-(2-nitrobenzoic acid)). DTNB is a form of TNB dimerized through disulfide bonds. The thiol readily reacts with DTNB and cleaves the disulfide bond to produce TNB. TNB is easily ionized in aqueous solution and eventually displays a yellow color. TNB can be quantified by measuring the change in absorbance at 412 nm using 14,150 M^-1^cm^-1^ as an extinction coefficient in diluted buffer solution. In this study, absorbance was measured using a DU730 UV/Vis spectrophotometer (Beckman Coulter) and 100 μL micro quartz cuvette.

### 2.5 Measurement of M2 peptide oxidation kinetics

Metal ion-dependent oxidation kinetics of the M2 peptide were measured by preparing 4 tubes with 1mL of 60 μM M2 peptide in pH 7.4 phosphate buffer solution. Next, 10 molar equivalents of Zn(II), Ni(II) and Co(II) ions or control buffer were added to each M2 peptide, respectively, and each metal ion-containing M2 peptide solution together with the control sample was oxygen-bubbled for 10 minutes and then incubated in an O_2_-saturating environment in a glove bag. Then, 90 μL samples were taken at 0, 1, 2, 4, 6, and 22 hours and mixed with 10 μL of 5 mM DTNB. The DTNB reaction was allowed to proceed for 10 minutes, and the change in absorbance at 412 nm was determined by subtracting the sample absorption spectrum from that of buffer mixed DTNB solution.

### 2.6 Determination of alkylation rate constant of M2 peptide

The metal ion dependence on alkylation rate was measured by mixing M2 peptide with iodoacetamide. Briefly, 1.5 mL of 30 μM M2 peptide was prepared in 20 mM PBS buffer (pH 8.0) at room temperature, after which 3 molar equivalents of TCEP were added to reduce any pre-formed inter- or intra-disulfide bonds. Alkylation reaction was initiated by adding 150 μL 10 mM iodoacetamide in H_2_O, after which 150 μL from the reaction mixture was withdrawn at each designated time point (0, 0.5, 1, 2, 3, 4, and 5 minutes, with additional time points of 10, 20, and 30 minutes for Zn(II)-added M2 peptide). For the metal-peptide complex experiment, 10 molar excess Zn(II) or Ni(II) ions were added prior to initiation of the alkylation reaction and incubated for at least 30 minutes. The alkylation reaction was then quenched by adding 95% formic acid and dithiothreitol. Finally, the reaction product was analyzed using a C18-HPLC and the observed kinetic profile was fitted by linear regression to yield the observed rate constant. For the determination of alkylation rate constants, triplicate experiments were carried out.

### 2.7 Measurement of HM2 antibody oxidation kinetics

Metal ion-dependent oxidation kinetics were measured for HM2. Briefly, 5 mL aliquots of HM2 at 10 μM in 20 mM phosphate buffer (20 mM sodium phosphate, 0.3 M NaCl, pH 7.4) were prepared. Next, 10 molar equivalents of TCEP were added to reduce disulfide bonds and the samples were incubated at 4°C for 30 minutes. The TCEP-reduced HM2 was subjected to oxygen-bubbling for 5 minutes, then divided into two tubes each containing 2.5 mL. One tube then received 10 molar equivalent Zn^2+^, while an equivalent volume of phosphate buffer was added to the other tube as a control. Next, 90 μL was taken from each tube and 10 μL of 5 mM DTNB (5,5’-dithiobis-(2-nitrobenzoic acid)) was added. After incubation at room temperature for 10 minutes, the absorbance change at 412 nm was measured. Absorbance measurements were repeated after incubation for 1, 2, 4, 6, and 22 hours in an oxygen environment.

### 2.8 AlexaFluor488-maleimide conjugation to HM2 and trastuzumab antibodies

Site-specific conjugation of HM2 utilizing Zn(II) ion was investigated by preparing 15 μM HM2 and Trastuzumab in 10 mM sodium succinate buffer with 30 mM sucrose (pH 6.0). Samples were then reduced with 45 μM (final concentration) TCEP for 30 minutes at 4°C, after which the excess TCEP was removed by dialysis against 10 mM sodium succinate buffer (pH 6.0 with 30 mM sucrose) containing 60 μM Zn(II) ions. The free cysteine contents of HM2 and trastuzumab were measured by monitoring the change in absorbance at 412 nm during the dialysis process using DTNB reaction. When the free cysteine contents reached 2, the reduced HM2 and trastuzumab were treated with 15 μM AlexaFluour488-maleimide and then incubated for 1 hour at room temperature. The un-reacted dye was quenched by adding excess (10 molar equivalent) L-cysteine, then removed by four rounds of extensive centrifugation filtration at 13,000 rpm for 5 minutes each.

### 2.9 IdeS cleavage of HM2 and trastuzumab

Site-specific conjugation was investigated by utilizing IdeS to identify the conjugation site. Briefly, 100 μg of protein was mixed with 1 μL of IdeS protease and 20 μL of 10× buffer, followed by dilution with 79 μL of distilled water. The dye-conjugated antibodies were treated with IdeS to identify the conjugation site. The cleavage reaction proceeded for 2 hours, after which the final product was analyzed by 12% SDS-page. A fluorescence image of the dye-conjugated protein was analyzed using a Typhoon fluorescence image analyzer (Amersharm Bioscience, USA). The image was further evaluated using the accompanying ImageQuant software (Amersham Bioscience, USA, emission filter, Alexafluor; PMT voltage parameter, 430V; sensitivity, normal; pixels, 25 microns).

## Results

### 3.1 Metal binding of M1 peptide (ACGHA)

**Fig 1:**
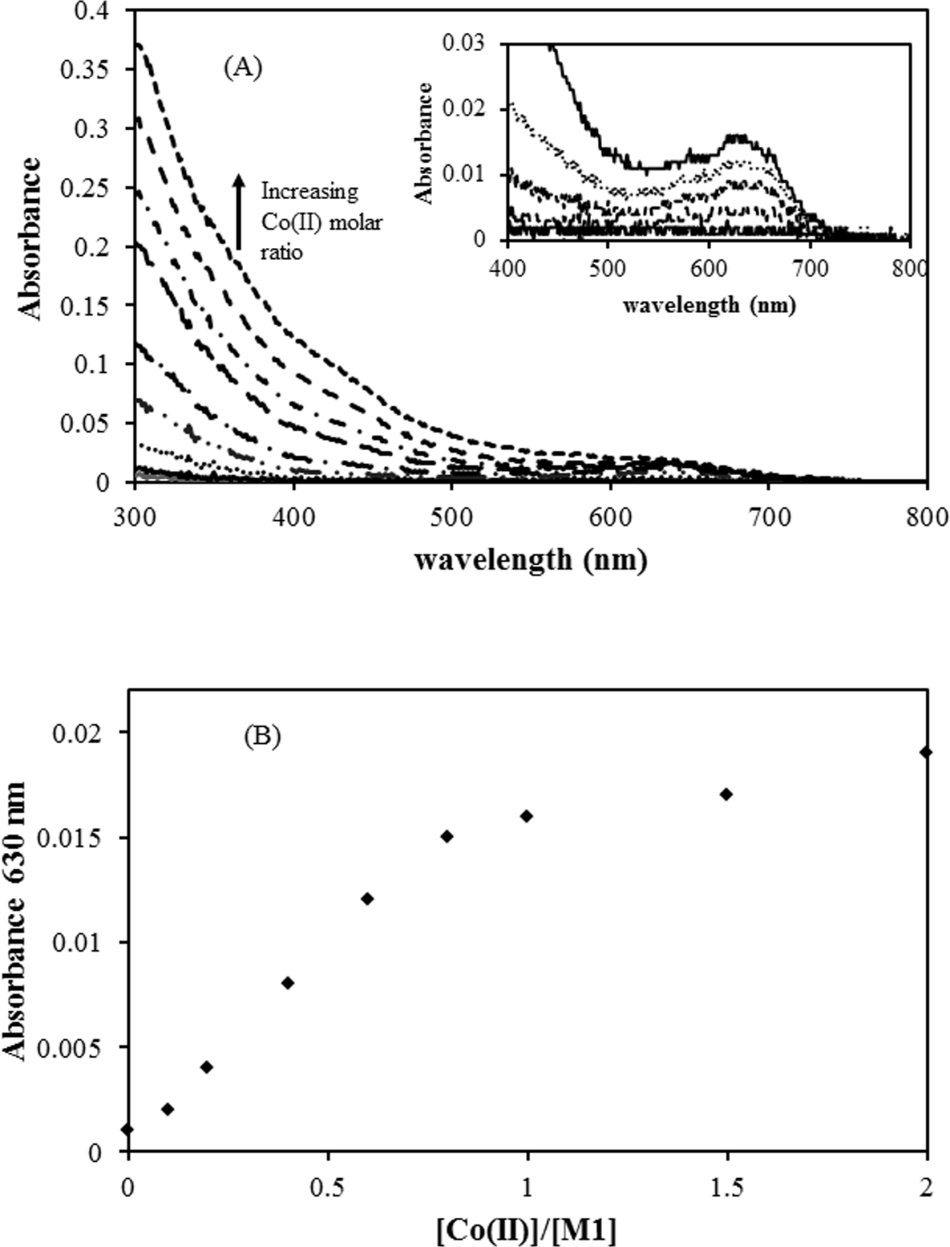
(A) The change of absorption spectra of Co(II)-bound M1 peptide (400 μM in pH 7.4 20 mM phosphate buffer) upon stepwise Co(II) addition (inset; the widened view of the absorption spectra near 630 nm region). (B) The Co(II)-binding isotherm of M1 peptide was obtained by monitoring changes in absorbance at 630 nm.

We prepared a pentapeptide (M1), (Ac)ACGHA, containing the CGH motif according to the study conducted by Burrows et al [27]. M1 peptide contains a single CGH motif with an acetylated N-terminus and a free carboxylic acid at the C-terminus. Burrows et al. reported a pH-dependent change in the coordination environment in the CGH motif. N-terminal acetylation removes the complexity associated with pH-dependent changes in the coordination environment of the CGH motif and reflects the possible amino acid environment when it is introduced into a protein. We conducted a Co(II) binding titration experiment for M1 peptide. M1 peptide was prepared at 400 μM in pH 7.4, 20 mM phosphate buffer and Co(II) binding to the ACGHA motif produced broad absorption spectra from the UV region to as far as 750 nm with a characteristic peak shoulder at 630 nm (Fig 1(A)). The extinction coefficient at 630 nm was calculated to be 50 M^-1^ cm^-1^ and is assumed to be a Co(II) absorption band. The Co(II) binding isotherm at 630 nm revealed that the metal binding stoichiometry was 1:1 binding (Fig 1(B)). When we introduced a serine mutation replacing cysteine, the Co(II) binding capability was abrogated, indicating that thiol groups of cysteine are critical to the metal binding pocket in the CGH motif (data not shown). In the CGH motif with N-acetylation, two alpha nitrogens, one from the histidine side chain and one thiol from the cysteine, could constitute the metal coordination environment [27]. Therefore, in the serine-substituted peptide, 4 atom coordination environments could not be formed in the SGH motif, resulting in decreased binding affinity to Co(II).

### 3.2 The metal binding experiment of M2 peptide (ACGHAACGHA)

**Fig 2:**
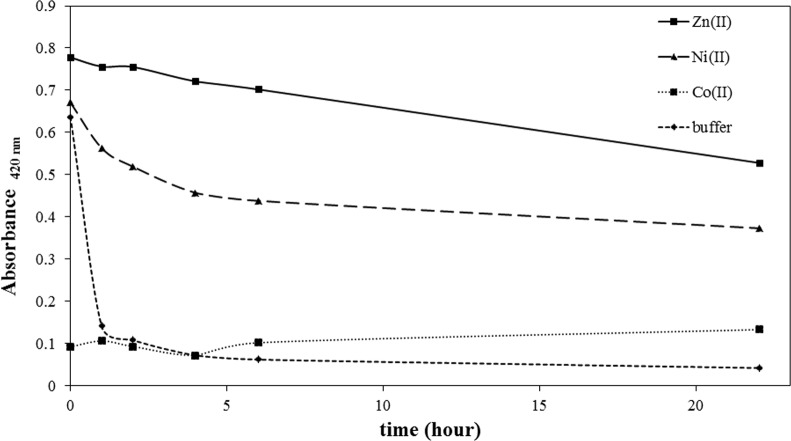
Co(II)-binding isotherm of M2 peptide (200 μM in pH 7.4, 20 mM phosphate buffer) was obtained by monitoring changes in absorbance at 360 nm upon stepwise Co(II) addition. Two molar equivalent Co(II) ion binding to a unimolar M2 peptide was observed. The sigmoidal binding isotherm suggests a concerted binding mode of Co(II) to M2 peptide. Inset, change in absorbance spectra of Co(II)-coordinated M2.

We prepared a peptide consisting of tandem repeat of CGH motifs and investigated the effects of a tandem array of metal binding motif on the metal binding characteristics and metal ion-dependent alkylation efficiency. M2 peptide consists of 10 amino acids, (Ac)ACGHAACGHA, with N-terminal acetylation and free C-terminal carboxylic acid. In the Co(II) binding experiment, M2 exhibited a 1:1 binding stoichiometry. Considering that a CGH motif could act as an independent metal chelating unit, and metal chelators consisting of 7–8 amino acids are too big to provide an adequate size for a single metal binding pocket, this binding stoichiometry was unexpected. One possibility of this observation is the formation of inter- or intra-peptide disulfide bonds and addition of 2 molar equivalents of TCEP produced 2:1 binding stoichiometry in Co(II) binding experiment to M2 peptide, as is shown in Fig 2. TCEP is known to be a poor metal chelator, and a control experiment of TCEP binding with Co(II) did not produce an observable change in Co(II) absorption spectra (data not shown). Moreover, the addition of extra TCEP to M2 did not change the binding stoichiometry of M2 peptide toward Co(II), indicating that TCEP itself does not participate in Co(II) binding (data not shown). The Co(II)-binding isotherm of TCEP-reduced M2 peptide presented in Fig 2 displayed an S-shaped binding curve, suggesting a concerted Co(II) binding mode. The first metal ion binding to M2 might trigger the conformational change in M2, eliciting a stout metal binding pocket for the second Co(II) binding.

### 3.3 Metal ion dependence on M2 peptide oxidation kinetics

**Fig 3:**
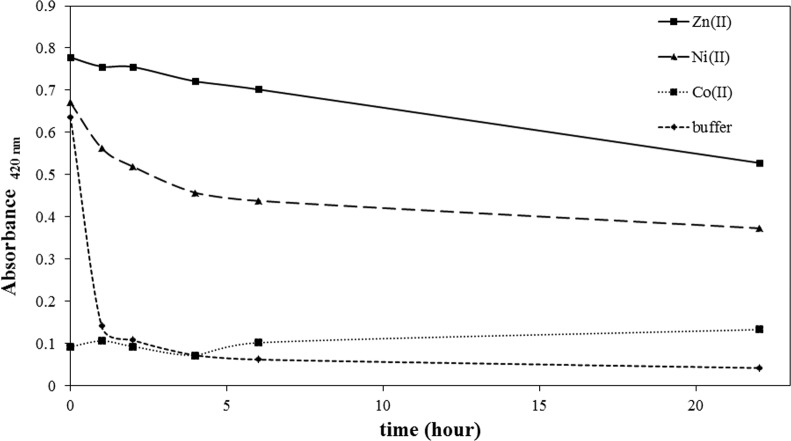
Metal-ion dependent oxidation kinetics of M2 peptide. Ten molar equivalents of Zn(II), Ni(II), and Co(II) ions, respectively, were added to 60 μM M2 peptide in pH 7.4 phosphate buffer solution. The free thiol content was measured for each sample after incubation under accelerated oxidation condition.

We investigated the metal-ion dependent oxidation kinetics in M2 peptide. The level of oxidation was measured by determining free thiol concentration utilizing the DTNB reaction. M2 peptide, which was prepared in pH 7.4, 20 mM phosphate buffer, was saturated with O_2_ by oxygen bubbling for 10 minutes. Without extraneous addition of oxidizing agent, disulfide formation through cysteine oxidation reaction proceeds very slowly, taking from several hours to days depending on the dissolved oxygen concentration. However, O_2_ saturation promotes oxidation reaction. As shown in Fig 3, M2 peptide, which does not contain any metal ions, showed a rapid oxidation kinetic profile and, within 1 hour, the free thiol content was shown to be near the baseline. Without metal ion protection, saturating oxygen conditions are assumed to accelerate the cysteine oxidation. M2 peptide shows a metal-ion dependence in oxidation kinetics, and metal ions protect M2 from oxidation in the order of Zn(II), Ni(II), and Co(II). Co(II) does not show any oxidation protection effect on M2 peptide under saturated oxygen conditions. When the free thiol content was measured immediately after oxygen bubbling, the level of Co(II)-bound M2 peptide is already reduced to a baseline. Ni(II) ion displayed a moderate oxidation protection effect and, interestingly, oxidation kinetic of Ni(II)-coordinated M2 showed a biphasic oxidation kinetic profile. Approximately 50% M2 showed rapid oxidation with a decay half-life of around 2 hours, followed by a slow oxidation reaction. The reason for this biphasic decay in the oxidation kinetic profile is not clear, but the difference between the two metal binding motifs observed with the Co(II) binding isotherm in M2 peptide might be associated with this biphasic decay. The loosely bound Ni(II) might account for the relatively fast decay in the oxidation kinetic profile. When compared with Co(II) or Ni(II) ions, Zn(II) displayed the highest oxidation protection efficiency. Zn(II) ions protected M2 from oxidation, and more than 70% M2 peptide retained its free cysteine reactivity after 22 hours.

### 3.4 Metal ion dependent alkylation kinetics of M2 peptide

**Fig 4:**
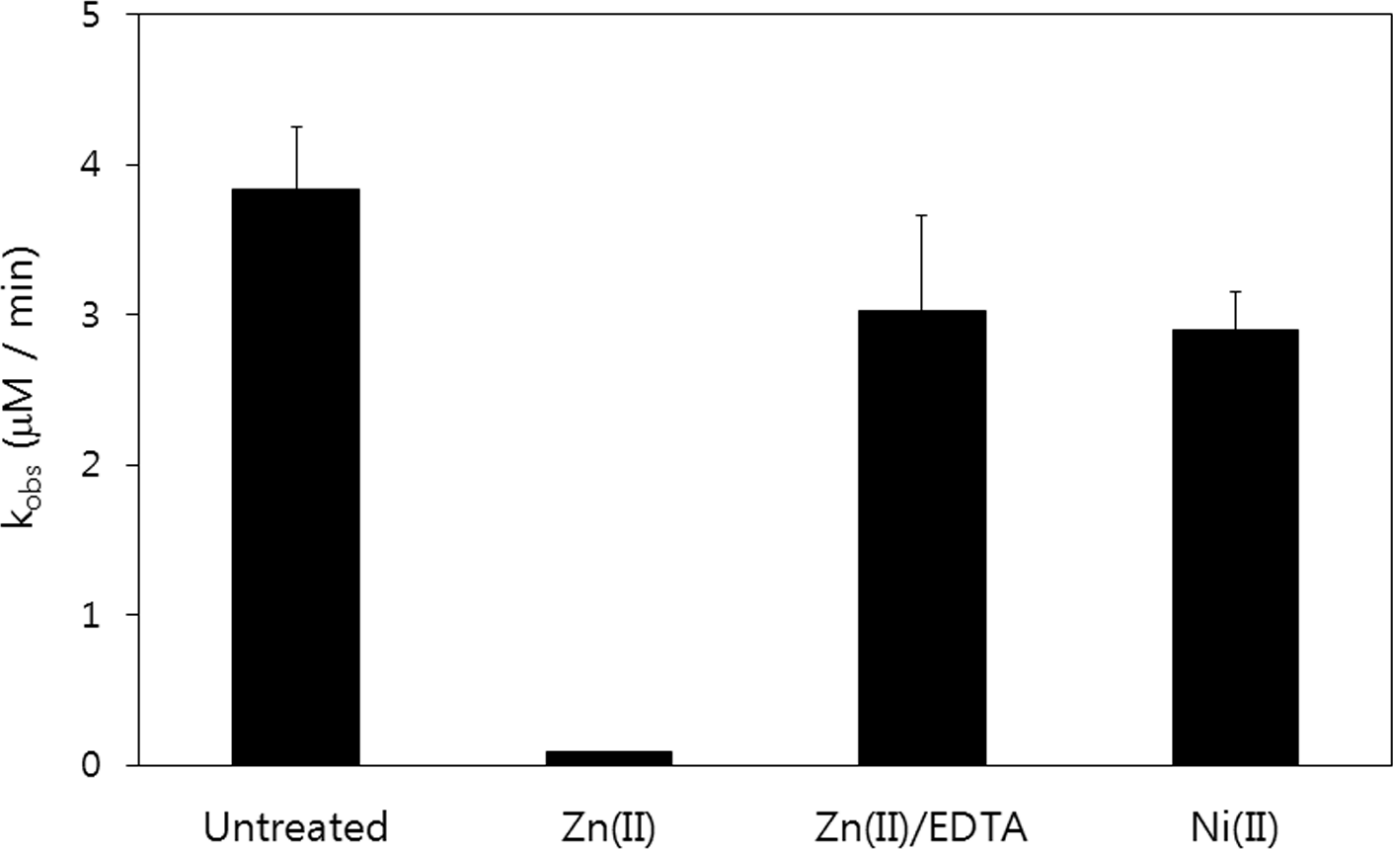
Observed rate constants for the alkylation of M2 peptides with iodoacetamide. Zn(II)-coordination significantly reduced the alkylation rate for M2 peptide and removal of coordinated Zn(II) with EDTA restored the alkylation rate.

Metal ion dependency was also observed in the alkylation reaction, and the alkylation reaction rate of M2 peptide was reduced significantly in the presence of Zn(II) ion as is shown in Fig 4. These findings are concordant with those reported by Burrows et al. for the M1 peptide [27]. Added metal ions affected thiol reactivity, and the observed rate constant of cysteine in M2 peptide toward iodoacetamide decreased by ~40 times in the presence of Zn(II) ions. The addition of excess EDTA to remove bound Zn(II) ions restored the alkylation reactivity in M2 peptide, indicating that Zn(II) binding is a factor inhibiting alkylation reaction. Ni(II) ion exhibited moderate inhibition reactivity, and in the presence of excess Ni(II) ions, the alkylation rate of M2 peptide was about 75% that of the apo-peptide. The difference in alkylation reaction rates between Zn(II) and Ni(II) is more prominent than that observed for oxidation protection kinetic profiles; however, the inhibitory effects still showed metal ion dependence in the order of Zn(II) > Ni(II).

We believe that the metal ion dependent differences in cysteine reactivity in M2 peptide toward oxidation and alkylation reaction could provide an invaluable tool in the context of site-selective conjugation. The slow oxidation reaction in the presence of Zn(II) ions could be used to selectively preserve cysteine reactivity in the metal binding motif under saturated oxygen conditions. The intrinsic cysteine residues that do not have metal binding properties could be oxidized ahead of the cysteine residues in the metal binding motif, thereby enabling site-selective conjugation at the cysteine residue of the metal binding motif under saturating metal ion conditions.

### 3.5 Metal ion dependent oxidation protection of the M2 antibody, HM2

As shown above, cysteine reactivity could be controlled by metal binding specificity. Therefore, we investigated the metal ion dependent oxidation protection in protein by introducing the metal binding motif into a protein. To accomplish this, we employed an antibody as a model system and there are several reasons for this. First, there are many intrinsic cysteine residues in antibody, as many as 16 in IgG1. Eight of these constitute four inter-chain disulfide bonds and the other eight are in four intra-chain disulfide bonds. Accordingly, antibodies are an ideal model system to evaluate the metal binding motif for the site-selective oxidation protection and subsequent conjugation scheme. Second, antibodies have been a major focus in antibody engineering for the last several decades, especially for application in antibody-drug conjugates [31,32]. Site-specific conjugation has recently attracted a great deal of interest in this field because of the need for homogeneity in the production of antibody-drug conjugates and the results of recent studies suggesting that site-specific conjugation is closely related to anti-cancer efficacy [33,34]. Therefore, in this study, the M2 peptide, ACGHAACGHA, was introduced at the C-terminus of trastuzumab antibody and the metal-ion dependent oxidation kinetics and site-specific alkylation were measured.

**Fig 5:**
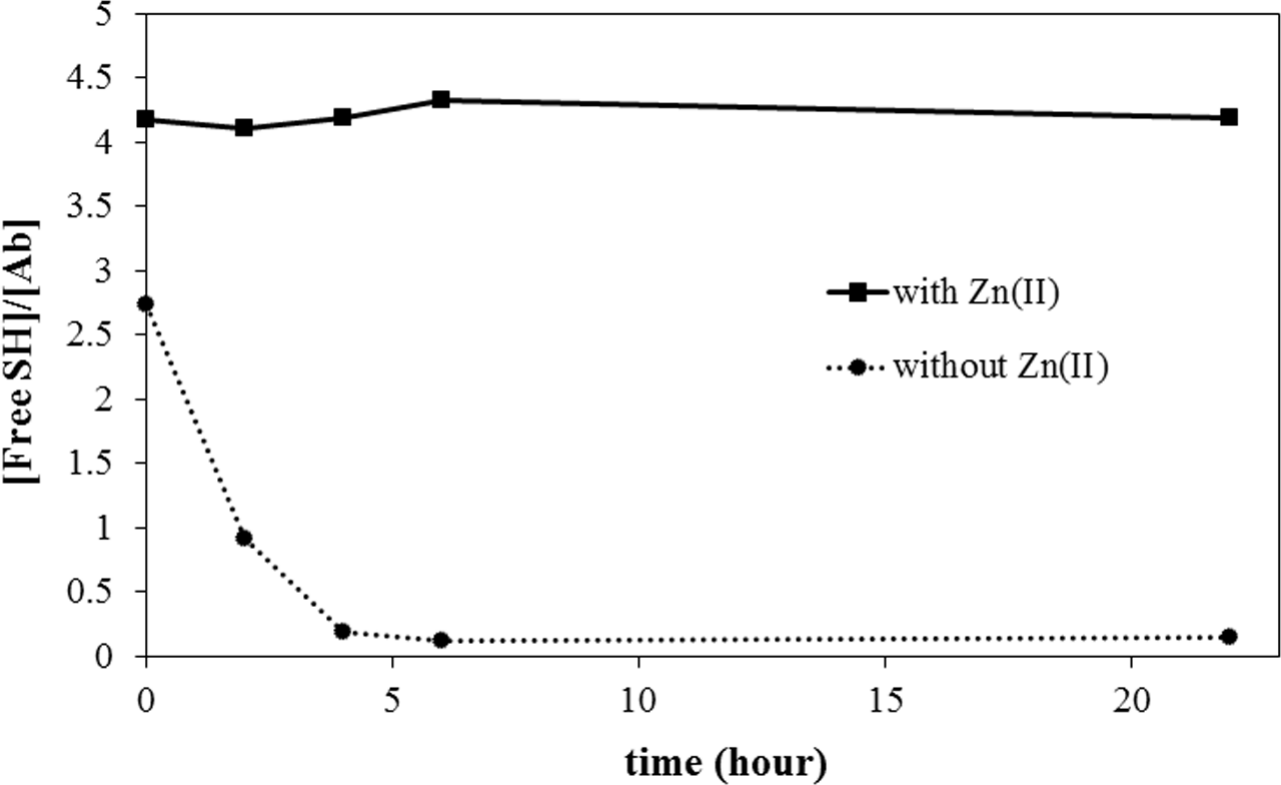
Zn(II)-ion dependent oxidation kinetics of M2 antibody (HM2) with (solid line) and without (dotted line) Zn(II) ions.

Metal-ion dependent oxidation kinetics were measured for M2-peptide-introduced trastuzumab (HM2). As shown in Fig 5, HM2 also showed Zn(II) ion dependent oxidation kinetics. Without added Zn(II) ion, the free thiol content of HM2 exhibited a rapid decay profile, with a half-life of approximately 1 hour. In addition, almost all free cysteines lost their reactivity in HM2 after 5 hours. In the presence of Zn(II) ion, the free thiol groups retained their reactivity until 22 hours after oxygen bubbling. Interestingly, approximately 4 free thiols were found in HM2 in the presence of Zn(II) ions, while about 2.7 were found without Zn(II) immediately after oxygen bubbling. As the introduction of M2 peptide at the C-terminus of heavy chain produced four metal binding motifs, each containing a single cysteine residue, four cysteine residues could be protected from oxidation in the presence of Zn(II) ions. As observed in the M2 peptide, without added metal ions, the cysteine residues were oxidized quickly under saturated oxygen conditions, so the measured free thiol content was much lower than the number of added cysteine residues (4). Moreover, the oxidation kinetics in HM2 also exhibited metal ion dependence in the order of Zn(II) > Ni(II) (data not shown) as observed in M2 peptide.

### 3.6 Zn(II)-dependent fluorophore binding to HM2

**Fig 6:**
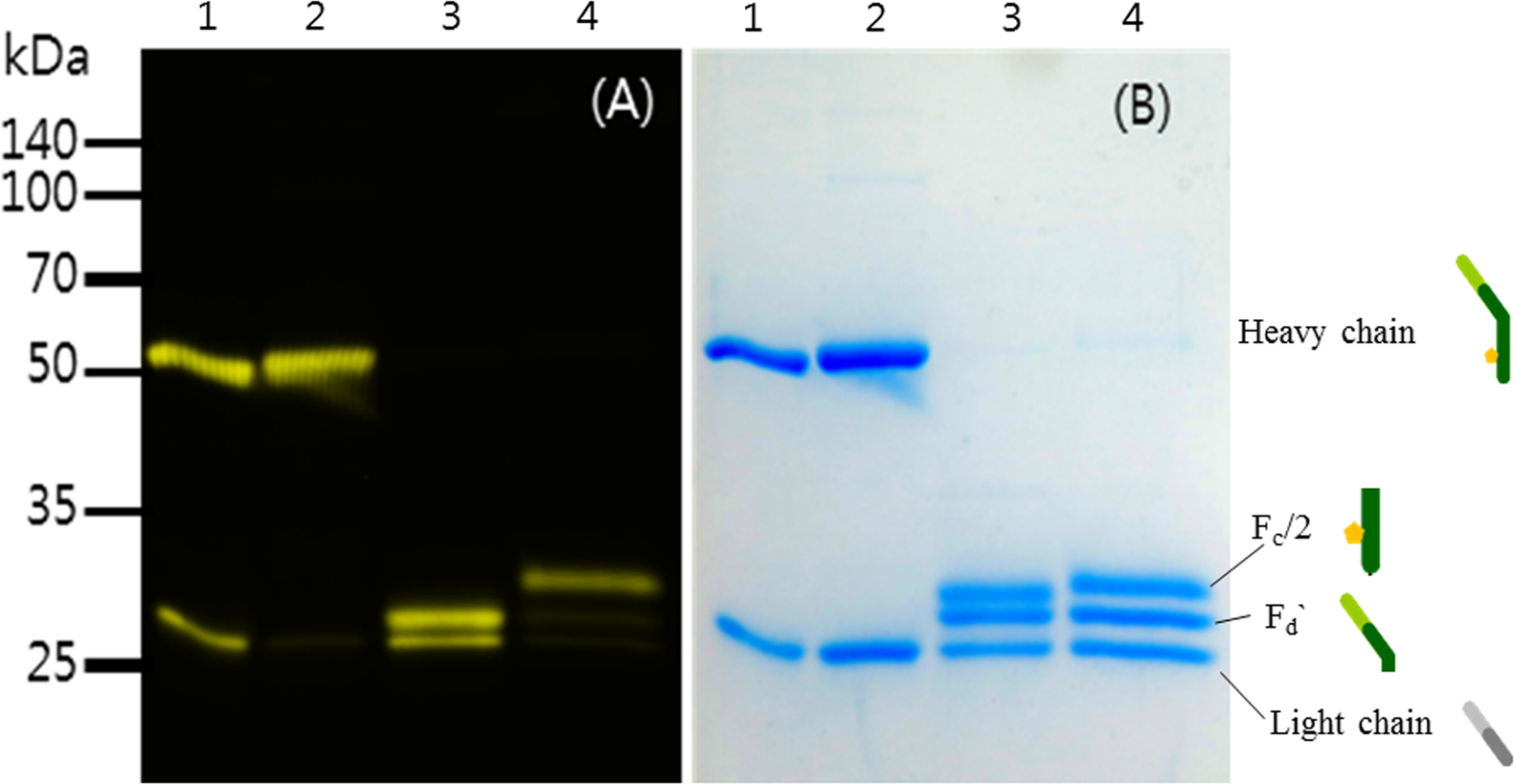
(A) The fluorescence scanning image of Alexaflour488-conjugated HM2 and AlexaFlour488-conjugated trastuzumab and (B) its reducing SDS-PAGE gel (12%) image. Lane 1: Alexafluor488-conjugated trastuzumab, Lane 2: Alexafluor488-conjugated HM2, Lane 3: IdeS treated Alexafluor488-conjugated trastuzumab, Lane 4: IdeS treated Alexafluor488-conjugated HM2.

Zn(II) ion-dependent dye conjugation reaction was studied with HM2 relative to trastuzumab, which does not have a metal binding motif. In the case of trastuzumab without metal binding motif, dye was conjugated site-nonspecifically to the reduced cysteine residues (to both heavy and light chains (Lane 1 in Fig 6(A)). Analyzing the conjugation percentile of trastuzumab revealed that the relative fluorescence intensities of trastuzumab measured for heavy and light chains were almost the same as those measured for the band intensities of heavy and light chains in the SDS-page gel (Lane 1 of Fig 6(B)). These findings indicated that added alexafluor488 dye was conjugated non-specifically to both chains. IdeS protease-cleaved trastuzumab indicated that Alexafluor488 dye was conjugated to light chain and Fd`fragments (Lane 3 in Fig 6(A)), while no fluorescence intensity was observed from fragmented Fc. Fluorescence intensity analysis of HM2 indicated that dye was predominantly (almost 95%) conjugated at the heavy chain (Lane 2 in Fig 6(A)). IdeS protease cleavage further confirmed the site-specific conjugation, with dye predominantly conjugated at fragmented Fc (Lane 4 in Fig 6(A)).

## Discussion

In this study, we investigated the potential applicability of the cysteine-containing metal binding motif for site-specific conjugation strategy. As reported by Burrow et al. [27], the metal binding peptide motif, ACGHA, provides an efficient coordination environment for metal ions. Replacing the cysteine residue with serine abrogated the metal binding efficiency of this peptide, indicating that the cysteine side chain plays a critical role in metal binding. We also designed a tandem repeat of metal binding motif, ACGHAACGHA, and investigated its metal binding characteristics. In the Co(II) binding experiment of the M1 peptide, the extinction coefficient of Co(II) at 630 nm was determined to be 50 M^-1^ cm^-1^, which was ascribed to the d-d transition of the Co(II) ion. The high absorption bands in the UV regions are assumed to reflect the ligand-to-metal charge transfer (LMCT) band [35]. This LMCT band is attributed to the electron transfer from the cysteine-thiolate to Co(II) ions and was not observed in the A**S**GHA peptide. In the Co(II) binding to M2 peptide, the d-d transition in the visible region showed a higher extinction coefficient than that of the M1 peptide, with ε = 150 M^-1^ cm^-1^ observed at 630 nm. The M2 peptide displayed a 1:2 (M2:Co(II)) stoichiometric binding isotherm with a concerted binding mode. The first Co(II) binding to M2 peptide might convert the peptide into a tighter binding coordination sphere for the second Co(II) ion. These results suggest that the tandem repeat of the metal binding motif in the M2 peptide could provide a tighter metal coordination sphere than the M1 peptide.

The metal-ion dependent oxidation kinetics of the M2 peptide exhibited metal-ion dependency. Metal ion displayed an oxidation-protection effect in the order of Zn(II) > Ni(II) > Co(II) as was observed for M1 peptide by Burrow et al. [27]. Measurement of the free cysteine content with excess Zn(II) ion in M2 peptide showed that cysteine residues in M2 peptide still retain their free thiol reactivity after 20 hours under accelerated oxidation conditions. Zn(II) ion also protected the cysteine thiol group from alkylation. With Zn(II)-saturating M2 peptide, the alkylation rate is much slower than that of M2 peptide without metal ions. Ni(II)-saturated M2 also displayed a slower alkylation rate than apo M2 peptide. Moreover, the alkylation protection effect displayed metal ion dependency in the order of Zn(II) >> Ni(II) > buffer.

The metal-ion dependent oxidation retardation behavior of the CGH motif could be exploited for site-specific conjugation to a protein. This site-specific conjugation strategy utilizing the metal binding motif might be very useful, especially for proteins that have many intrinsic cysteine residues. As cysteine residues are usually employed in structure-sustaining disulfide bonds, conjugation at the intrinsic cysteines could be problematic. To avoid this problem, cysteine residues could be introduced at various positions in the protein that do not compromise its structure. However, it is still not clear how to induce selective conjugation at the introduced cysteine residue. We believe that differences in the oxidation kinetics of the ligand-protected metal binding motif from other cysteines could provide an answer to this question. Upon Zn(II) binding, oxidation of the cysteine residue in the CGH metal binding motif could be prohibited for 22 hours or more. Accordingly, this conjugation strategy employing the metal-binding motif should proceed as follows: (1) reduction of disulfide cysteine, (2) metal ion binding to specifically protect the cysteine in metal binding motif, (3) oxidation to restore the unprotected cysteine to disulfide, (4) conjugation at the cysteine residue of the metal binding motif. For bound metal ions, the conjugation reaction might proceed slowly depending on conjugation conditions such as temperature and time. However, we found that prolonged incubation for 1–2 hours at room temperature resulted in a conjugation yield comparable to that of the metal ion-removed state. Without metal ion binding to selectively control the oxidation kinetics of cysteine residues, free thiol contents decrease promptly upon addition of oxidizing reagent for both intrinsic cysteines and the introduced metal binding motif. Following this conjugation scheme, we verified that the fluorescent dye could be selectively conjugated to the introduced cysteine residues at the C-terminus of the antibody. The introduced metal binding motif in the C-terminus of the antibody also showed Zn(II) dependent protection from oxidation and alkylation. The dye-conjugation experiment demonstrated that the metal binding motif could provide an efficient site-selective conjugation strategy, and Alexfluor488 dye was predominantly conjugated at the introduced C-terminus of HM2.

We believe that this site-specific conjugation strategy employing the metal binding motif is especially useful for antibody-drug conjugates. There have been several site-selective conjugation strategies to selectively label highly cytotoxic payloads onto antibodies, including enzymatic conjugation [36], bridging conjugation interconnecting the intrinsic disulfide bond with bidentate functional groups [37], non-natural amino acids [38,39], etc. Enzymatic conjugation methods utilize highly specific enzyme reactions, and enzyme recognition sites are inserted into target protein structures. The insertion sites are usually dictated by sequence and structural analysis, although the C-terminal end could be utilized to minimize the perturbation of antibody structures. Highly specific enzyme conjugation ensures the site-specificity, but the necessity for rather harsh reaction conditions [36] or a large excess of enzyme [40] casts doubt on the availability of this conjugation strategy for downstream process development. Bridging conjugation methods utilizes the existing inter-chain disulfide bonds of antibody. In this conjugation method, the bidentate functional group interconnects the inter-chain disulfide bond through bridge formation. Bridging conjugation exploits the pre-existing disulfide bond, and payload conjugation is expected to leave the inter-chain disulfide bond intact. However, competition between bridging formation and single-dentate conjugation to the reduced cysteine residues could produce a mixture of conjugation products [37], lowering the net production yield. Site-specific conjugation utilizing non-natural amino acids is based on the genetic control of tRNA synthetase to recognize chemically modified tRNA. This sophisticated conjugation strategy has been developed to incorporate non-natural amino acids into desired amino acid sites in antibodies. Chemically modified non-natural amino acids could provide an orthogonal conjugation site. However, this conjugation strategy necessitates the addition of non-natural ingredients during the culture process and laborious processes to induce genetic mutations.

We believe that site-selective conjugation strategies employing metal-binding motifs could be advantageous to other site-selective conjugation methods in several respects. This method provides an efficient and simple site-selective conjugation strategy that enables discrimination of engineered cysteine motifs from intrinsic ones based simply on their metal ion binding properties. The simple conjugation method with highly productive site-specific conjugation has great potential for use in the development of antibody drug conjugates. Selective installation of cytotoxic molecules at a pre-determined position with a pre-defined number of molecules could lead to a homogeneous product, which is beneficial for pharmacokinetic and therapeutic properties. We also believe that this simple and productive conjugation strategy has great potential for application in other areas in which site-specific conjugation is needed.

## References

[1] Kalia J, Raines RT. Advances in bioconjugation. Curr. Org. Chem. 2010; 14: 138–147. 20622973 10.2174/138527210790069839 PMC2901115

[2] Ismail NF, Lim TS. Site-specific scFv labelling with invertase via Sortase A mechanism as a platform for antibody-antigen detection using the personal glucose meter. Sci. Rep. 2016; 19(6):1–13. 10.1038/srep19338 PMC4726117 26782912

[3] Shein SA, Nukolova NV, Korchaqina AA, Abakumova TO, Kiuznetsov II, Abakumova MA, et al Site-directed delivery of VEGF-targeted liposomes into intracranial C6 glioma. Bull. Exp. Biol. Med. 2015; 158(3):371–376. 10.1007/s10517-015-2765-4 25573371

[4] Liu Y, Pierce R, Luehmann HP, Sharp TL, Welch MJ. PET imaging of chemokine receptors in vascular injury-accelerated atherosclerosis. J. Nucl. Med. 2013; 54(7):1135–1141. 10.2967/jnumed.112.114777 23658218 PMC4251467

[5] Li L, Olafsen T, Anderson A-L, Wu A, Raubitschek AA, Shively JE. Reduction of kidney uptake in radiometal labeled peptide linkers conjugated to recombinant antibody fragments. Site-specific conjugation of DOTA-peptides to a Cys-diabody. Bioconjug. Chem. 2002; 13:985–995. 12236780 10.1021/bc025565u

[6] Lewis MR, Kao JY, Anderson A-LJ, Shively JE, Raubitschek A. An improved method for conjugating monoclonal antibodies with N-hydroxysulfosuccinimidyl DOTA. Bioconjug. Chem. 2001; 12:320–324. 11312695 10.1021/bc0000886

[7] Wang L, Yuan L, Wang H, Liu X, Li X, Chen H. New strategy for reversible modulation of protein activity through site-specific conjugation of small molecule and polymer. Bioconjug. Chem. 2014; 25:1252–1260. 10.1021/bc5000934 24971741

[8] Hermanson G.T. Bioconjugate technuques 2^nd^ Ed(London, UK; Academic Press, 2008). pp. 169–192.

[9] Watanabe M, Fukada H, Inoue H, Ishikawa K. Crystal structure of an acetylesterase from Talaromyces cellulolyticus and the importance of a disulfide bond near the active site. FEBS Lett. 2015; 589(11):1200–1206. 10.1016/j.febslet.2015.03.020 25825334

[10] Gasymov OK, Abduragimov AR, Glasgow BJ. Restoration of structural stability and ligand binding after removal of the conserved disulfide bond in tear lipocalin. Biochem. Biophys. Res. Commun. 2014; 452(4):1004–1008. 10.1016/j.bbrc.2014.09.029 25223802 PMC4219327

[11] Urade Y, Tanaka T, Eguchi N, Kikuchi M, Kimura H, Toh H, et al Structural and functional significance of cysteine residues of glutathione-independent prostaglandin D synthase. Identification of Cys65 as an essential thiol. J. Bio. Chem. 1995; 270(3):1422–1428. 7836410 10.1074/jbc.270.3.1422

[12] Web B, Lampe JN, Roberts AG, Atkins WM, David Rodrigues A, Nelson SD. Cysteine 98 in CYP3A4 contributes to conformational integrity required for P450 interaction with CYP reductase. Arch. Biochem. Biophys. 2006; 454(1):42–54. 16959210 10.1016/j.abb.2006.08.003 PMC2001172

[13] Wang H, He L, Lensch M, Gabius HJ, Fee CJ, Middelberg AP. Single-site Cys-substituting mutation of human lectin galectin-2: modulating solubility in recombinant production, reducing long-term aggregation, and enabling site-specific monoPEGylation. Biomacromolecules 2008; 9(11):3223–3230. 10.1021/bm800801b 18942878

[14] Finney LA, O’Halloran TV. Transition metal speciation in the cell: insight from the chemistry of metal ion receptors. Science 2003; 300(5621):931–936. 12738850 10.1126/science.1085049

[15] Nies DH, Brown NL. Metal ions in gene regulation, (New York, Chapman & Hall, 1998). pp 77–103.

[16] Hanas JS, Hazuda DJ, Bogenhagen DF, Wu FY, Wu CW. Xenopus transcription factor A requires zinc for binding to the 5S RNA gene. J. Biol. Chem. 1983; 258(23):14120–14125. 6196359

[17] Krishna SS, Majumdar I, Grishin NV. Structural classification of zinc finger: survey and summary. Nucleic Acids Res. 2003; 31(2):532–550. 12527760 10.1093/nar/gkg161 PMC140525

[18] Singh VK, Xiong A, Usgaard TR, Chakrabarti S, Deora R, Misra TK, et al ZntR is an autoregulatory protein and negatively regulates the chromosomal zinc resistance operon znt of Staphylococcus aureus. Mol. Microbiol. 1999; 33(1):200–207. 10411736 10.1046/j.1365-2958.1999.01466.x

[19] Lee S, Arunkumar AI, Chen X, Giedroc DP. Structural insights into homo- and heterotropic allosteric coupling in the zinc sensor S. aureus CzrA from covalently fused dimers. J. Am. Chem. Soc. 2006; 128:1937–1947. 16464095 10.1021/ja0546828

[20] Ohrvik H, Wittung-Stafshede P. Identification of new potential interaction partners for human cytoplasmic copper chaperone Atox1: roles in gene regulation? Int. J. Mol. 2015; 16(8):16728–16739. 10.3390/ijms160816728 PMC4581165 26213915

[21] Brautigam L, Johansson C, Kubsch B, McDonough MA, Bill E, Holmgren A, et al An unusual mode of iron-sulfur-cluster coordination in a teleost glutaredoxin. Biochem. Biophys. Res. Commun. 2013; 436(3):491–496. 10.1016/j.bbrc.2013.05.132 23756812

[22] Xie F, Sutherland DE, Stillman MJ, Ogawa MY. Cu(I) binding properties of a designed metalloprotein. J. Inorg. Biochem. 2010; 104(3):261–267. 10.1016/j.jinorgbio.2009.12.005 20060593

[23] Peacock AFA, Iranzo O, Pecoraro VL. Harnessing natures ability to control metal ion coordination geometry using de Novo designed peptides. Dalton Trans. 2009; 13:2271–2280. 10.1039/b818306f 19290357 PMC3046812

[24] Plegaria JS, Dzul SP, Zuiderweg ERP, Stemmler TL, Pecoraro VL. Apoprotein structure and metal binding characterization of a de Novo designed peptide, α3DIV, that sequesters toxic heavy metals. Biochemistry 2015; 54:2858–2873. 10.1021/acs.biochem.5b00064 25790102 PMC4492461

[25] Stanyon HF, Cong X, Chen Y, Shahidullah N, Rossetti G, Dreyer J, et al Developing predictive rules for coordination geometry from visible circular dichroism of copper(II) and nickel(II) ions in histidine and amide main-chain complexes. FEBS J. 2014; 281(17):3945–3954. 10.1111/febs.12934 25039600

[26] Griep MA, Adkins BJ, Hromas D, Hohnson S, Miller J. The tyrosine photophysics of a primase-derived peptide are sensitive to the peptide’s zinc-bound state: proof that the bacterial primase hypothetical zinc finger sequence binds zinc. Biochemistry 1997; 36(3):544–553. 9012670 10.1021/bi9612778

[27] van Horn JD, Bulaj G, Goldenberg DP, Burrows CJ. The Cys-Xaa-His metal-binding motif: {N} versus {S} coordination and nickel-mediated formation of cysteinyl sulfinic acid. J. Biol. Inorg. Chem. 2003; 8:601–610. 12827456 10.1007/s00775-003-0454-7

[28] Lu H, Zhang H, Chen J, Zhang J, Liu R, Sun H, et al A thiol fluorescent probe reveals the intricate modulation of cysteine’s reactivity by Cu(II). Talanta 2016; 146:477–482. 10.1016/j.talanta.2015.09.014 26695293

[29] Kassim R, Ramseyer C, Enescu M. Oxidation reactivity of zinc-cysteine clusters in metallothionein. J. Biol. Inorg. Chem. 2013; 18(3):333–342. 10.1007/s00775-013-0977-5 23334196

[30] Ellman G.L. Tissue sulfhydryl groups. Arch. Biochem. Biophys. 1959; 82(1):70–77. 13650640 10.1016/0003-9861(59)90090-6

[31] Barok M, Tanner M, Koninki K, Isola J. Trastuzumab-DM1 is highly effective in preclinical models of HER2-positive gastric cancer. Cancer Lett. 2011; 306:171–179. 10.1016/j.canlet.2011.03.002 21458915

[32] Zinzani PL, Pellegrini C, Cantonetti M, Re A, Pinto A, Pavone V, et al Brentuximab Vedotin in transplant-naïve relapsed/refractory Hodgkin lymphoma: experience in 30 patients. Oncologist 2015; 20(12):1413–1416. 10.1634/theoncologist.2015-0227 26500229 PMC4679088

[33] Junutula JR, Flagella KM, Graham RA, Parsons KL, Ha E, Raab H, et al Engineered thio-trastuzumab-DM1 conjugate with an improved therapeutic index to target human epidermal growth factor receptor 2-positive breast cancer. Clin. Cancer Res. 2010; 16(19):4769–4778. 10.1158/1078-0432.CCR-10-0987 20805300

[34] Shen BQ, Xu K, Liu L, Raab H, Bhakta S, Kendrick M, et al Conjugation site modulates the in vivo stability and therapeutic activity of antibody-drug conjugates. Nat. Biotechnol. 2012; 30(2):184–189. 10.1038/nbt.2108 22267010

[35] McMillin DR, Rosenberg RC, Gray HB. Preparation and spectroscopic studies of cobalt(II) derivatives of blue copper proteins. Proc. Nat. Acad. Sci. USA 1974; 71:4760–4762. 4216022 10.1073/pnas.71.12.4760 PMC433976

[36] Drake PM, Albers AE, Baker J, Banas S, Barfield RM, Bhat AS, et al Aldehyde tag coupled with HIPS chemistry enables the production of ADCs conjugated site-specifically to different antibody regions with distinct in vivo efficacy and PK outcomes. Bioconjug. Chem. 2014; 25(7):1331–1341. 10.1021/bc500189z 24924618 PMC4215875

[37] Smith MEB, Schumacher FF, Ryan CP, Tedaldi LM, Papaioannou D, Waksman G, et al Protein modification, bioconjugation, and disulfide bridging using bromomaleimides. J. Am. Chem. Soc. 2010; 132:1960–1965. 10.1021/ja908610s 20092331 PMC2842020

[38] Zhang Z, Smith BAC, Wang L, Brock A, Cho C, Schultz PG. A new strategy for the site-specific modification of proteins in vivo. Biochemistry 2003; 42:6735–6746. 12779328 10.1021/bi0300231

[39] Bullock TL, Rodriguez-Hernandez A, Corigliano EM, Perona JJ. A rationally engineered misacylating aminoacyl-tRNA synthetase. Proc. Natl. Acad. Sci. USA 2008; 105(21):7428–7433. 10.1073/pnas.0711812105 18477696 PMC2396676

[40] Strop P, Liu SH, Dorywalska M, Delaria K, Dushin RG, Tran TT, et al Location matters: site of conjugation modulates stability and pharmacokinetics of antibody drug conjugates. Chem. Biol. 2013; 202(2):161–167. 10.1016/j.chembiol.2013.01.010 23438745

